# Modified iliac screw technique for pelvic fixation: a scoping review of technical characteristics and early clinical outcomes

**DOI:** 10.1007/s00701-026-06850-2

**Published:** 2026-04-01

**Authors:** Hanyu Qiu, Dhruvish Patel, Moriah Thompson, Piper Tingleaf, Kishore Balasubramanian, Peter G. Passias, Luis M. Tumialan, Praveen V. Mummaneni, Nitin Agrawal, Ali K. Ozturk, Hakeem J. Shakir, John F. Burke, Chao Li, Zachary A. Smith, Andrew Jea, Angela E. Downes, M. Burhan Janjua

**Affiliations:** 1 Department of Neurosurgery, University of Oklahoma College of Medicine, 1000 N. Lincoln Blvd., Ste. 4000, Oklahoma City, OK 73104 USA; 2 College of Medicine, Texas A&M Health Science Center, Houston, TX USA; 3 Department of Neurosurgery and Orthopedic Surgery, Duke University School of Medicine, Durham, NC USA; 4 Department of Neurosurgery, Barrow Neurological Institute, St. Joseph’s Hospital and Medical Center, Phoenix, AZ USA; 5 Department of Neurological Surgery, University of California San Francisco Medical Center, San Francisco, CA USA; 6 Department of Neurosurgery, University of Pittsburgh Medical Center, Pittsburgh, PA USA; 7 Department of Neurosurgery, University of Pennsylvania Hospital, Philadelphia, PA USA; 8 Department of Neurosurgery, Texas Health Physicians Group, Arlington, TX USA

**Keywords:** Modified iliac screw, Lumbosacral fusion, Spinopelvic fixation, Spinal deformity

## Abstract

**Introduction:**

Iliac screws remain essential in cases requiring spinopelvic fixation, providing distal anchorage and construct stability across many surgical indications. However, traditional posterior-superior-iliac–spine (PSIS) entry screws are associated with hardware prominence, postoperative pain, and the need for side-to-side connectors to link with primary rods. The S2 alar-iliac (S2AI) technique addresses these drawbacks but some series have reported higher mechanical failure rates due to acute screw angulation. Furthermore, the S2AI trajectory violates the sacroiliac (SI) joint, potentially limiting use in cases with compromised sacral anatomy. The modified iliac screw (MIS) technique was developed to overcome these issues by shifting the entry point medial to the PSIS, maintaining iliac purchase while avoiding SI joint involvement. This scoping review synthesizes early clinical usage and implant performance of this emerging technique.

**Methods:**

A scoping review following PRISMA guidelines was conducted across PubMed, MEDLINE, and Embase through Sep 28, 2025. Eligible studies included reports describing patient outcomes following fixation using modified iliac screws.

**Results:**

A total of 162 patients across 6 studies were included. The cohort had a mean age of 64.5 years (range; 18–78). The most common indication was deformity (62.3%; *n* = 101), followed by fixation following tumor resection (17.9%; n = 29) and degenerative disease (9.3%; *n* = 15). The mean number of fixed vertebrae was 8.01 (range; 3–18). Mean follow-up time was 19.19 months (range; 3–74). Implant complications were reported in 36 cases (22.2%), commonly screw perforation (*n* = 22), screw fracture (*n* = 5), and screw loosening (*n* = 4). Complications led to implant failure for 7 patient cases (4.3%).

**Conclusion:**

Early experiences with MIS fixation demonstrate promising clinical utility with acceptable rates of hardware-related complications and morbidity. However, existing evidence remains limited to small, heterogeneous series without standardized outcome reporting. Robust prospective investigations with larger cohorts and standardized long-term follow-up are needed to comprehensively characterize the safety, durability, and comparative performance of the MIS technique.

## Introduction

Achieving a reliable fusion at the lumbosacral junction remains one of the most difficult aspects of long-segment reconstruction in adult spinal deformity (ASD). High mechanical stress across L5–S1 predisposes this region to pseudarthrosis and instrumentation failure, which has led surgeons to extend fixation into the pelvis for added stability. Traditional iliac screws (TIS) are effective for achieving sacropelvic fixation, but a recent review highlights consistently high complication rates including mechanical failure, pseudarthrosis, sacroiliac joint pain, wound complications, and revision surgery, attributing many of these issues to the prominence and soft-tissue burden of the iliac screw head at the posterior superior iliac spine (PSIS) [11]. The S2 alar-iliac (S2AI) technique addresses many of these limitations, however, mechanical failures have been attributed to the acute angle between the screw head and shaft, and the trajectory necessarily traverses the sacroiliac (SI) joint which may limit its use in patients with compromised sacral or SI joint anatomy [1, 13, 22].

To reduce these complications, a medialized and low-profile modification of the traditional iliac screw trajectory, which also avoids the SI joint, has been developed. The modified iliac screw (MIS) technique shifts the starting point medially and caudally relative to the PSIS, toward the rudimentary S1–S2 region, while maintaining a trajectory toward the anterior inferior iliac spine (AIIS) [3]. This pattern positions the screw head deeper within the ilium and closer to the midline, providing a protective osseous ledge while improving soft-tissue coverage and often eliminating the need for offset connectors [3]. Cadaveric measurements confirm that the medialized entry increases the screw-to-skin distance and produces a lower-profile construct compared with the traditional PSIS entry point [3]. Complementary anatomical evaluations show that this modified iliac screw trajectory both remains safely intraosseous and maintains similar distances from key neurovascular structures such as the supragluteal bundle and greater sciatic notch relative to the traditional iliac screw technique [4].

Biomechanical studies support the construct validity of modified iliac screw fixation. Both cyclic loading cadaver models and finite-element analyses have reported comparable screw toggle, rod strain, and stress distribution between modified iliac screws, traditional iliac screws, and S2 alar-iliac (S2AI) fixation [5, 20]. Clinically, the more medial starting point aligns the iliac tulip with lumbar and sacral pedicle screws, simplifying rod insertion and reducing the need for aggressive contouring [18]. Early reported clinical experience with modified, subcrestal iliac screw techniques also suggests favorable outcomes [12]. The deeper screw-head position and improved alignment allowed most constructs to avoid side-connectors, highlighting one of the intended advantages of the technique [12]. Related deformity literature identifies offset connector use itself as an independent predictor of distal implant failure, emphasizing the importance of connector-free constructs [25].

Although current findings have appeared encouraging, evidence on modified iliac screws remains dispersed across cadaveric studies, computational modeling, and retrospective clinical series. This scoping review aims to synthesize the available literature to evaluate the safety, biomechanical performance, and clinical outcomes associated with modified iliac screw fixation relative to established pelvic fixation methods.

## Methods

### Literature review

A literature search was completed to identify all publications reporting clinical use of modified iliac screw pelvic fixation, in accordance with the PRISMA-ScR (Preferred Reporting Items for Systematic Reviews and Meta-Analyses extension for Scoping Reviews) checklist [15, 24]. Because modified iliac screw fixation is a relatively new and variably described technique with limited clinical outcome data, we elected a scoping review methodology to comprehensively map the existing literature and identify knowledge gaps, rather than to perform a quantitative meta-analysis.

PubMed, Embase, and MEDLINE databases were searched from inception to September 28, 2025, operating the Boolean full-text search [(“Modified" OR "Medial" OR "Medialized") AND ("Iliac Screw")]. All search results were exported to Rayyan, and duplicates were manually deleted.

### Study selection

Articles were included if they (1) included patients who underwent pelvic fixation using the modified iliac screw technique, (2) reported patient treatment and outcome data, and (3) were written in English. Studies were excluded if they (1) were autopsy reports, animal studies, or cadaver studies, (2) were literature reviews, meta-analyses, systematic reviews, perspectives, or editorials, and/or (3) were from non-English or non-peer reviewed sources.

Two independent reviewers (D.P. and M.T.) screened all titles and abstracts and then assessed the full texts of articles that met the inclusion criteria. A third reviewer (H.Q.) settled disagreements. Eligible papers were included.

### Data extraction and synthesis

One reviewer (H.Q.) extracted data from each article, which was then confirmed independently by two additional reviewers (D.P. and M.T.). Extracted data included study author, year published, patient demographics, clinical indication, treatment characteristics, and post-operative outcomes. The primary outcome of interest was the rate of hardware failure and postoperative morbidity associated with modified iliac screws compared with traditional iliac screw techniques.

All data was compiled and analyzed using Microsoft Excel (Microsoft Corporation, Redmond, WA, USA). Continuous variables are presented as weighted means with ranges, and categorical variables as frequencies with percentages.

### Data quality assessment

For each study, two independent authors (H.Q. and D.P.) assessed the risk of bias by applying the Joanna Briggs Institute checklists for case reports, case series, or cohort studies. The level of evidence of included studies was evaluated using the Joanna Briggs Institute Levels of Evidence guidelines.

## Results

### Study selection

**Fig. 1:**
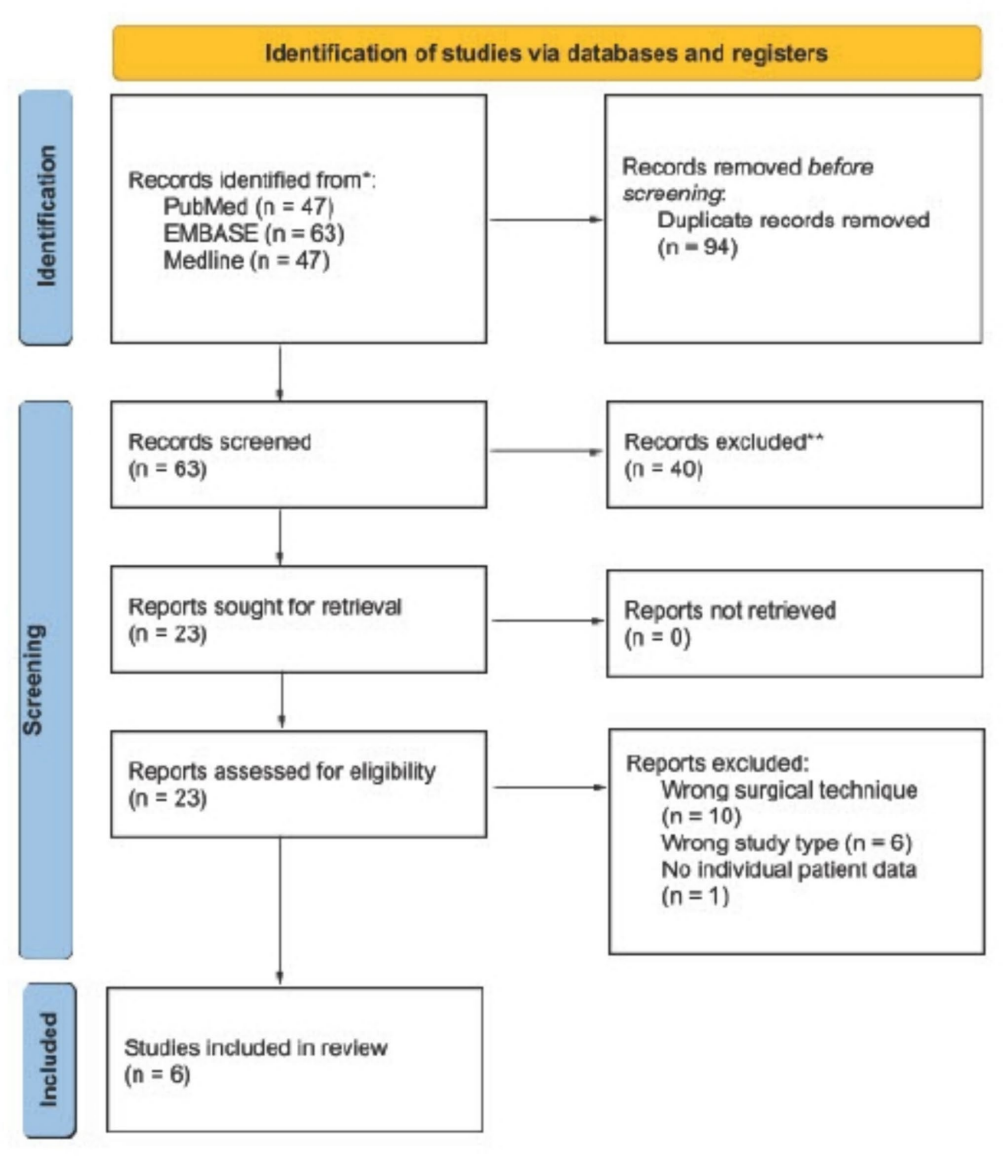
PRISMA flow diagram of the literature screening

Figure 1 summarizes the study selection process using a PRISMA flow diagram. The initial systematic search identified a total of 157 studies (PubMed: 47, EMBASE: 63, Medline: 47). After removal of 94 duplicates, 63 studies were screened using predefined inclusion and exclusion criteria. Finally, six studies were included in the final synthesis, which consisted of one cohort study, three case series, and two case reports, representing Joanna Briggs Institute (JBI) levels of evidence 3.c, 4.c, and 4.d, respectively [6, 9, 12, 21, 23, 27].

### Patient characteristics

The six included studies comprised a total of 162 patients undergoing lumbopelvic fixation with MIS. Individual study characteristics, including publication year, geographic location, study design, and level of evidence, are summarized in Table 1. The weighted mean age across the cohort was 64.5 years (range: 18–78 years), with 90 female patients (55.6%) and 72 male patients (44.4%) (Table 2).

**Table 1:** Summary of included studies

Study Author	Year	Study Type	JBI Level of Evidence	Study Location	Treatment Group	Number of Patients	Mean Age (yrs)	Sex	Mean Follow-up (months)	No. of Implant Related Complications (%)	No. of Patients with Implant Failure (%)
Huang et al	2023	Case series	4.c	China	MIS	27	52	F:9 M: 18	22.7	1 (4%)	0 (0%)
Zhang et al	2022	Case report	4.d	China	MIS	1	43	M	60	0 (0%)	0 (0%)
Von Glinski et al	2022	Cohort Study	3.c	Germany	MIS	113	67.6	F: 71 M: 42	16.32	31 (27%)	7 (6%)
					TIS*	40	70.2	F: 26 M:14	14.39	15 (38%)	4 (10%)
					S2AI*	37	64.8	F: 24 M: 13	11.93	9 (24%)	3 (8%)
Tanasaomboon et al	2021	Case report	4.d	Thailand	MIS	1	64	M	12	0 (0%)	0 (0%)
Liu et al	2017	Case series	4.c	Singapore	MIS	10	66.4	F: 5 M: 5	27.3	2 (20%)	0 (0%)
Sohn et al	2016	Case series	4.c	Korea	MIS	10	63.3	F: 5 M: 5	30.7	2 (20%)	0 (0%)

*Comparator arms (TIS, S2AI) are shown for context from von Glinski et al.; pooled demographics/outcomes reflect MIS patients only

*MIS* modified iliac screw, *TIS *traditional iliac screw, *S2AI* S2 alar-iliac

**Table 2:** Summary of patient characteristics

Characteristic (no. of patients for whom information is available)	*N *or Mean*	Range or %
Cohort size (no.)	162	
Patient age (*n* = 162)		
Age (years)	64.5	18–78
Patient sex (*n* = 162)		
Female	90	55.6
Male	72	44.4
Clinical indication (*n* = 162)		
Spinal deformity	101	62.3
Tumor/neoplasm	29	17.9
Degenerative	15	9.3
Infection/inflammation	9	5.6
Trauma/fracture	8	4.9
Location of pathology/injury (*n* = 31)		
S1-S5	29	93.5
L1-L5	2	6.5

*Represents weighted mean across included studies

Indications for surgery were most commonly spinal deformity (*n* = 101, 62.3%), followed by tumor or neoplastic pathology (*n* = 29, 17.9%), degenerative disease (*n* = 15, 9.3%), infection or inflammatory conditions (*n* = 9, 5.6%), and trauma or fracture (*n* = 8, 4.9%). Among patients with reported vertebrae pathology levels (*n* = 31), pathology/injury most frequently involved the sacral levels S1–S5 (*n* = 29, 93.5%), followed by the lumbar spine (L1–L5) (*n* = 2, 6.5%).

### Treatment characteristics

Across the included studies, the mean number of spinal segments fixed was 8.01 levels (range: 3–18). The mean duration of follow-up was 19.19 months (range: 3–74 months) (Table 3). Implant-related complications were reported in 36 patients (22.2%). The most common complication was screw perforation (*n* = 22, 13.6%), followed by screw fracture (*n* = 5, 3.1%) and screw loosening (*n* = 4, 2.5%) (Table 3). Implant failure was uncommon, with 155 patients (95.7%) demonstrating no evidence of implant failure during follow-up. Reported failures included screw fracture in five patients (3.1%), screw pullout in one patient (0.6%), and rod fracture at the lumbopelvic fixation site in one patient (0.6%).

**Table 3:** Summary of treatment and outcomes

Treatment Characteristic (no. of patients for whom information is available)	*N* or Mean*	Range or %
Number of segments fixed (*n* = 162)	8.01	3–18
Follow-up (months) (*n* = 162)	19.19	3–74
MIS Implant complications (*n* = 162)		
None	126	77.8
Screw perforation	22	13.6
Screw fracture	5	3.1
Screw loosening	4	2.5
Unspecified	3	1.9
Screw prominence	1	0.6
Rod fracture at LPF	1	0.6
Hardware failure (*n* = 162)		
No implant failure	155	95.7
Screw fracture	5	3.1
Screw pullout	1	0.6
Rod fracture at LPF	1	0.6

*Represents weighted mean across included studies

*MIS* modified iliac screw, *LPF* lumbopelvic fixation

## Discussion

Effective pelvic fixation remains essential for long posterior constructs, yet the optimal strategy continues to involve tradeoffs between construct durability, soft-tissue morbidity, and anatomic constraints. While traditional iliac screw (TIS) fixation provides effective sacropelvic anchorage, it is limited by soft-tissue irritation, screw head prominence, and the need for offset connectors to align with the primary rod and the cephalad pedicle screw construct [21]. To address these limitations, sacral alar-iliac (S2AI) screws were developed with a deeper, more medial starting point that reduces implant prominence and often avoids the need for offset connectors [3, 16]. However, S2AI fixation also traverses the sacroiliac (SI) joint, which may pose an anatomic limitation of the technique [26]. Furthermore, some studies have suggested increased rates of mechanical complications with S2AI fixation compared to traditional iliac screws [2, 7]. In light of these considerations, the modified iliac screw (MIS) technique has emerged as an alternative fixation strategy aimed at providing stable pelvic fixation while reducing screw head prominence and avoiding SI joint violation [3, 21]. Given the preliminary and heterogeneous nature of the current literature, firm conclusions cannot be drawn, and the findings presented in this review serve as a descriptive synthesis intended to guide future investigation rather than establish definitive comparative outcomes.

### Overview of technique

**Fig. 2:**
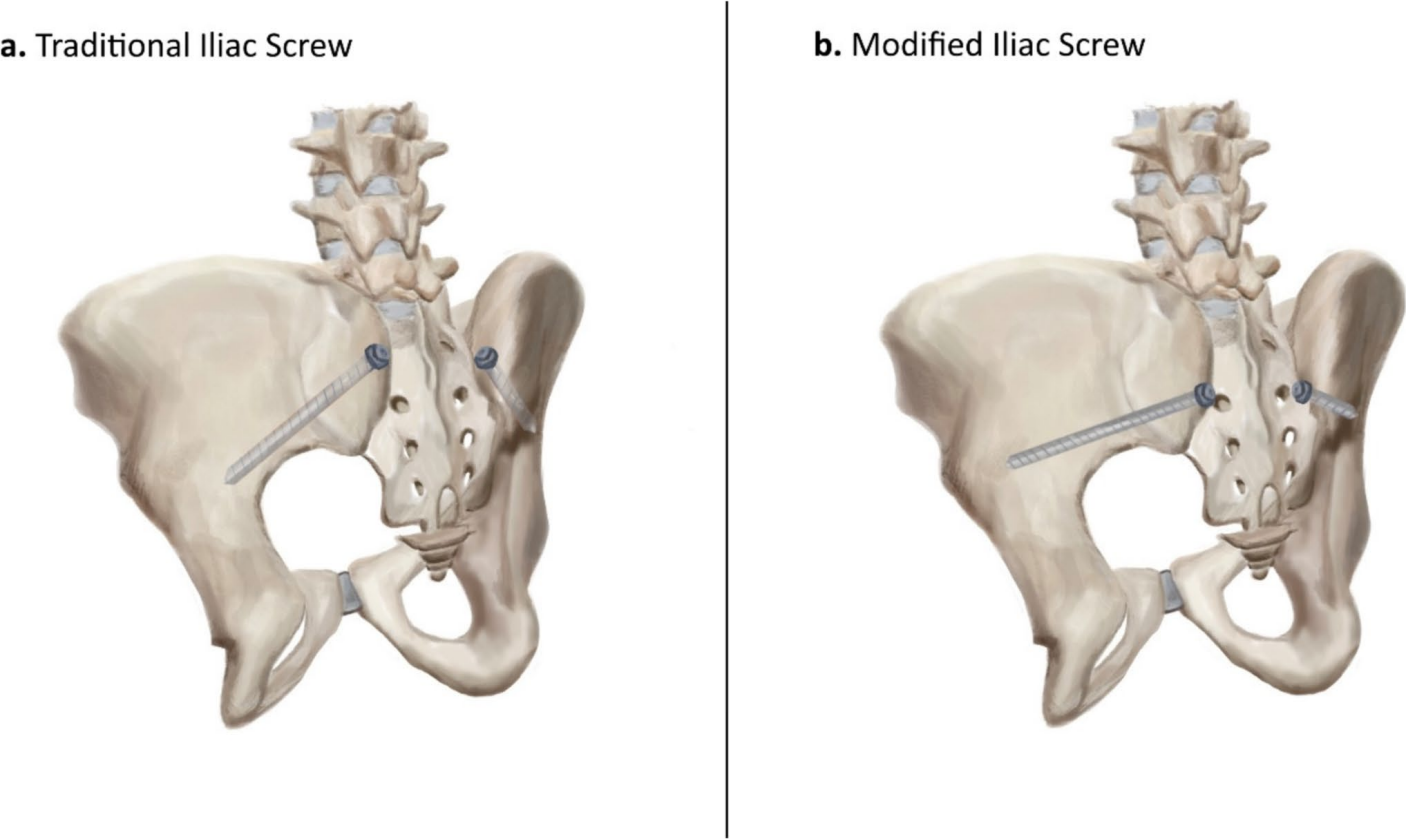
Illustration of pelvic model with iliac screws. **a** Traditional iliac screw (TIS). **b** Modified iliac screw (MIS). *Original illustration created in-house by the authors (P.T.)*

Methods for pelvic fixation have evolved considerably over time, shifting from early instrumentation methods such as the Galveston technique toward modern screw-based pelvic fixation constructs, typically using the posterior superior iliac spine (PSIS) as an entry point with a trajectory towards the anterior inferior iliac spine (AIIS) [4, 17]. This PSIS to AIIS corridor has been well characterized and remains a widely established reference trajectory for iliac screw placement, offering known stability and clinical utility [4]. However, traditional iliac screw constructs are limited by potentially symptomatic hardware prominence and connector-related interfaces required to align the iliac screws with the cephalad rod system [5]. The modified iliac screw technique aims to retain the favorable osseous purchase and fixation stability of traditional iliac screws, while medializing and caudalizing the entry point to reduce hardware prominence and facilitate inline construct engagement [3]. The modified iliac screw (MIS) technique retains a trajectory directed towards the AIIS but shifts the starting point 1.5 cm caudal and 1.0 cm medial from the traditional PSIS entry, allowing the screw head to align with the S1 pedicle screw and avoiding additional connectors (Fig. 2) [3].

**Fig. 3:**
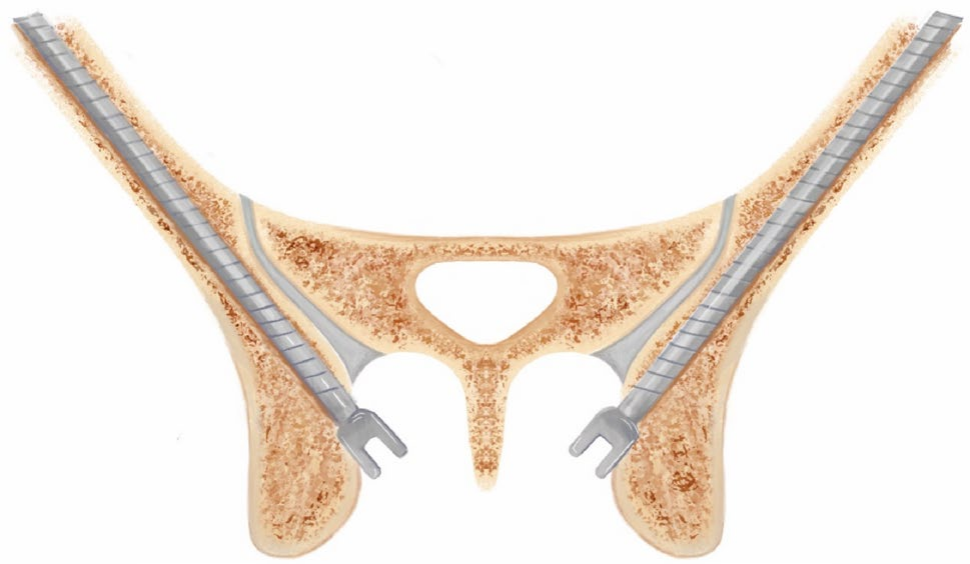
Cross-section of pelvic model with modified iliac screw (MIS). *Original illustration created in-house by the authors (P.T.)*

Recent cadaveric studies have provided empirical support for the technical premise of modified iliac screw (MIS) fixation [3, 5]. In a 12 cadaver comparison reported by von Glinski et al., MIS increased the mean screw-head to skin distance compared with traditional iliac screws (TIS), with mean distances of 2.4 cm (range, 1.2–4.2 cm) for TIS and 3.2 cm (range, 1.7–4.3 cm) for MIS [3]. MIS also reduced the screw-head lateral offset from midline, with a mean offset of 3.1 cm (range, 2.4–4.5 cm) compared with 4.2 cm (range, 3.7–4.9 cm) for TIS [3]. Together, these measurements correspond to an approximate 23% increase in soft-tissue coverage depth and a 24.5% reduction in midline offset with MIS compared with TIS, aligning with the technique’s goals of reducing symptomatic prominence and reducing the need for offset connectors (Fig. 3) [3].

### Biomechanical findings

A dynamic loading cadaver study was also performed by von Glinski et al. to evaluate construct stability with MIS fixation in comparison to more established techniques including TIS and S2AI [5]. 18 specimens (6 per technique) underwent pedicle and pelvic screw fixation. Each specimen was cyclically loaded at ± 10 Nm for 50,000 cycles at a frequency of 3 Hz, with the average screw toggle and rod strain in the last 1,000 cycles recorded [5]. The results indicated that S2AI pelvic screws exhibited the most screw toggle relative to the pelvis, with MIS showing the least screw toggle, while TIS was in between [5].

Additionally, rod strain was also tested, with values taken from both above and below the S1 pedicle screw. Above S1, total strain was highest with MIS constructs, followed by TIS constructs and S2AI constructs [5]. The trend was reversed when rod strain was measured below the S1 pedicle screw, with S2AI group exhibiting the greatest strain, followed by TIS and MIS [5]. However, differences in rod strain were not statistically significant between tested construct groups, supporting comparable mechanical behavior under the tested cyclic loading conditions [5].

**Fig. 4:**
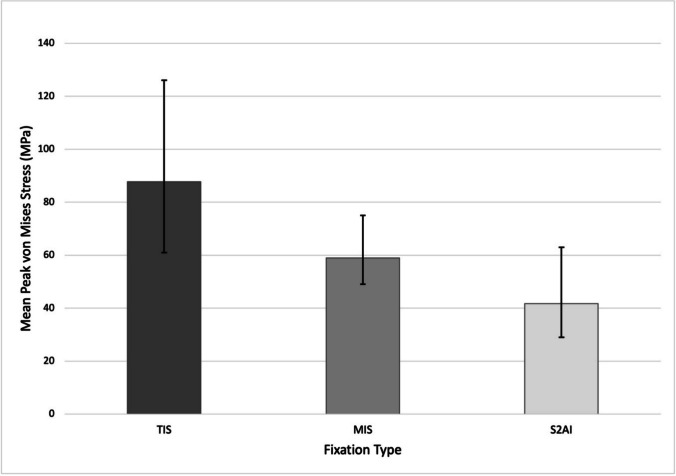
Mean PVMS (MPa) values across tested motion states. Bars represent the unweighted mean PVMS across flexion, extension, lateral bending, and axial rotation. Error bars indicate the range of PVMS values observed across motion states. *Graph created by the authors using values from Sohn *et al*. * [20]

Further biomechanical evidence comes from finite element analysis performed by Sohn et al., where peak von Mises stress (PVMS) and stress localization were compared for MIS, TIS, and S2AI based L1-to-pelvis constructs [20]. Tested motion states included flexion, extension, lateral bending, and axial rotation. Overall, PVMS was highest for the TIS group across tested motion states, with S2AI demonstrating the lowest overall PVMS while MIS exhibited an intermediate result [20]. Mean PVMS values across tested motion states are summarized in Fig. 4.

Differing screw head-shaft interaction force directionality was observed, with S2AI demonstrating a predominantly distractive interaction at the head–shaft interface and MIS demonstrating predominantly compressive interaction at the head–shaft junction [20]. The authors suggested that while S2AI exhibited lower PVMS than MIS, the stress loading pattern in S2AI was a factor that may contribute to screw-head deformation and technique-specific mechanical failure patterns [20]. Nonetheless, all PVMS values across constructs remained below fatigue strength thresholds under physiologic loading conditions [20].

Taken together, the cadaveric cyclic-loading analysis and finite element stress analysis provide a coherent biomechanical rationale for MIS fixation and support construct-level mechanical behavior that is broadly comparable to established pelvic fixation strategies under the tested conditions [5, 20]. These biomechanical findings provide a framework for interpreting the early clinical outcomes and complication profiles reported in the available series.

### Clinical outcomes and considerations

Pelvic fixation is clinically used across a wide range of indications, commonly including spinal deformity correction, reconstruction following tumor removal, and trauma/fractures [8]. The available early literature on MIS-based pelvic fixation reflects the broader clinical indication landscape, with the most common indications in our cohort being spinal deformity (62.3%) and reconstruction after tumor removal (17.9%). Across all included clinical reports, modified iliac screw fixation was performed using a bilateral construct consisting of one MIS screw per side, reflecting a consistent pelvic fixation strategy across the available literature. Within this setting, early reports of MIS clinical application have shown encouraging findings when contextualized with technique goals and biomechanical characteristics.

A recent meta-analysis found an implant failure rate of 18.9% (95% CI, 11.4%−26.6%) for S2AI screws, and a failure rate of 21.9% (95% CI, 14.0%−26.4%) for TIS screws [19]. Our early cohort demonstrated lower reported failure rates, with only 7 out of 162 cases (4.3%) reporting implant failure. However, implant-related complications were reported in 36 cases (22%), with the majority of cases not progressing to failure requiring revision surgery. The primary complication of our MIS cohort involved iliac wing screw perforation (*n* = 22, 13.6%). In the studies reporting this complication, the findings were reported as asymptomatic radiographic cortical breaches detected on postoperative computed tomography (CT) imaging and were not associated with neurovascular or visceral injury [6, 9]. While revision surgery was not required in reported screw perforation cases, definitions of cortical breach direction and magnitude varied, with long-term functional and mechanical impact remaining uncertain from the existing clinical literature. It is important to note that these data derive from small early clinical reports, and substantially more evidence from larger cohorts with long-term follow-up will be required to determine a true rate of complication and failure.

When observing the patterns of morbidity associated with MIS fixation, within the limitations of heterogeneous early evidence, the trends appear to align with the favorable aspects of S2AI constructs, while contrasting with the typical morbidity concerns of TIS-based pelvic fixation. Meta-analyses and retrospective studies comparing S2AI and traditional iliac screw techniques demonstrate that traditional iliac screw fixation carries significantly higher risks of postoperative complications involving symptomatic screw prominence and wound issues than S2AI fixation, along with concerns regarding sacroiliac (SI) pain [10, 11, 19]. Our MIS cohort displayed low rates of screw head prominence, with only one case (0.6%) reported. No MIS cases reported postoperative SI joint pain; however, specific SI symptom reporting was inconsistent across studies, with only one case series explicitly documenting the absence of postoperative SI joint symptoms [12]. Nonetheless, the early observed morbidity profile suggests that the modified iliac screw trajectory with increased soft-tissue coverage may translate into clinically relevant symptomatic improvement for patients.

While the more medial entry point of S2AI pelvic fixation has typically translated to clinically relevant improvements to prominence-associated morbidity, previous publications have identified concerns regarding higher mechanical failure possibility, potentially from acute screw-head to screw-shaft angulation [13]. Common modes of reported failures include screw loosening and rod or set-screw displacement from the screw head in S2AI constructs [13, 14]. Similar to S2AI fixation, the MIS trajectory employs a more medial and deeper entry point than traditional iliac screws, but unlike S2AI, MIS maintains a lateral iliac corridor without crossing the SI joint and follows a less steep screw angle [3, 8]. Regardless, the medialized entry of MIS does suggest corresponding concerns regarding screw head angulation, reflective of concerns with S2AI. While our MIS cohort’s rate of hardware related failures was within ranges reported in existing literature of established techniques, our data is limited by small sample size and short follow-up. Although MIS entry is lateral to the entry point of S2AI, screw head angulation should nonetheless be a factor clinicians are aware of, and future studies should place emphasis on examining this relationship and how it compares with known techniques.

Broadly, our results align well with what was observed in a cohort study by von Glinski et al. that compared MIS (*n* = 113), TIS (*n* = 40), and S2AI (*n* = 37) pelvic fixation [6]. The authors found that MIS fixation yielded the lowest rate of symptomatic hardware prominence (0% MIS, 7.5% TIS, 8.1% S2AI) [6]. Hardware failure was also lowest in the MIS group (6.2%) compared to 10% for the TIS group and 8.1% for the S2AI group [6]. Notably, 2 of the 4 hardware failures seen in the TIS group were from the offset connectors, a factor that is avoided with MIS and S2AI [6].

### Limitations

This study has several important limitations inherent to both the available literature and the scoping review methodology. Primarily, the clinical evidence base for modified iliac screw (MIS) fixation remains small and heterogeneous owing to the novelty of the technique and consists predominantly of retrospective case series and case reports, with one comparative cohort study. Clinical indications and construct characteristics were heterogeneous, with varying fixation lengths and patient characteristics that cannot be adequately adjusted for without standardized data. Furthermore, follow-up duration was limited and variable, with a weighted mean follow-up of approximately 19 months. Mechanical failure modes relevant to pelvic fixation such as late rod fracture and screw loosening may occur beyond this time frame. As a result, the pooled estimates of complication and failure rates should be interpreted as early signals rather than definitive benchmarks, particularly when compared with the much larger and more mature literature surrounding traditional iliac screw (TIS) and S2 alar-iliac (S2AI) fixation.

In addition, a substantial proportion of the pooled MIS cohort derives from a single cohort study, accounting for most included patients. As a result, the weighted complication and failure estimates presented in this review are heavily influenced by that study’s patient population and institutional experience. Accordingly, these aggregate rates should be interpreted with caution and may not be fully generalizable to broader clinical practice settings. Furthermore, standardized patient-reported outcome measures (PROMs) and quality-of-life metrics were not consistently reported across the included studies. As a result, conclusions regarding the patient-centered functional outcomes associated with MIS fixation cannot be reliably drawn from the current literature.

Finally, although cadaveric and finite element analyses provide valuable mechanistic insight, biomechanical performance under laboratory conditions does not guarantee translation to in vivo performance. As a scoping review, this study is not designed to establish comparative superiority. Rather, it highlights emerging clinical patterns and knowledge gaps. Prospective comparative studies with standardized outcome reporting and long-term follow-up are needed to more definitively define the clinical role, mechanical performance, and patient-centered impact of MIS fixation relative to established pelvic fixation strategies.

## Conclusion

Pelvic fixation remains a key component of spinal constructs across a wide range of clinical indications; however, the optimal strategy for distal anchorage remains unsettled, with existing techniques offering distinct advantages and tradeoffs. Within this landscape, modified iliac screw (MIS) fixation represents a clinically relevant alternative aimed at mitigating several limitations of traditional pelvic fixation trajectories.

MIS fixation constitutes a biomechanically coherent evolution of pelvic fixation, preserving the established iliac corridor purchase of traditional iliac screws while achieving a lower-profile construct without sacroiliac joint traversal or reliance on offset connectors. Across cadaveric, computational, and early clinical literature, MIS demonstrates mechanically coherent construct behavior and early reported outcomes; however, existing clinical reports remain preliminary and descriptive in nature and should not be interpreted in direct comparisons with established methods. Nonetheless, the observed complication profile has been broadly consistent with the intended soft-tissue and prominence-reducing goals of the modified iliac screw technique, although the available evidence base is limited by small sample sizes and short follow-up. Prospective, comparative investigations with standardized outcome reporting and long-term follow-up are needed to validate these observations and establish the durability, complication profile, and clinical role of MIS fixation within contemporary pelvic fixation strategies.

## Data Availability

All data generated or analyzed during this study is included in this article. Further inquiries may be directed to the corresponding author.
